# COVID-19 Detection Using Photoplethysmography and Neural Networks

**DOI:** 10.3390/s23052561

**Published:** 2023-02-25

**Authors:** Sara Lombardi, Piergiorgio Francia, Rossella Deodati, Italo Calamai, Marco Luchini, Rosario Spina, Leonardo Bocchi

**Affiliations:** 1 Department of Information Engineering, University of Florence, 50139 Florence, Italy; 2 Ospedale San Giuseppe, 50053 Empoli, Italy

**Keywords:** photoplethysmogram, microcirculation, deep learning, convolutional neural network, modelling, classification

## Abstract

The early identification of microvascular changes in patients with Coronavirus Disease 2019 (COVID-19) may offer an important clinical opportunity. This study aimed to define a method, based on deep learning approaches, for the identification of COVID-19 patients from the analysis of the raw PPG signal, acquired with a pulse oximeter. To develop the method, we acquired the PPG signal of 93 COVID-19 patients and 90 healthy control subjects using a finger pulse oximeter. To select the good quality portions of the signal, we developed a template-matching method that excludes samples corrupted by noise or motion artefacts. These samples were subsequently used to develop a custom convolutional neural network model. The model accepts PPG signal segments as input and performs a binary classification between COVID-19 and control samples. The proposed model showed good performance in identifying COVID-19 patients, achieving 83.86% accuracy and 84.30% sensitivity (hold-out validation) on test data. The obtained results indicate that photoplethysmography may be a useful tool for microcirculation assessment and early recognition of SARS-CoV-2-induced microvascular changes. In addition, such a noninvasive and low-cost method is well suited for the development of a user-friendly system, potentially applicable even in resource-limited healthcare settings.

## 1. Introduction

COVID-19 is an infectious respiratory disease caused by SARS-CoV-2, a coronavirus discovered in the city of Wuhan, China, in 2019 [1]. Since then, the virus has spread rapidly to other countries around the world, causing a global health and economic crisis. According to data from the World Health Organization (WHO), more than 664 million cases and more than 6.6 million deaths have been recorded as of January 2023 [2]. The rapid spread and the difficulties of treating patients with SARS-CoV-2 infection have led to the development of several diagnostic methods for the early recognition and treatment of patients with COVID-19. Except for the molecular test, based on reverse transcription-polymerase chain reaction (RT-PCR), which remains the reference diagnostic tool, low-cost and easy-to-perform procedures, and tests have also been proposed. Among these, the analysis of the photoplethysmogram (PPG) signal as a means for the early recognition of patients with COVID-19 in the hospital setting has been suggested.

COVID-19 infection typically presents with symptoms such as weakness or fatigue with fever, dry cough and shortness of breath. In severe infection, the symptomatology may progress to serious complications such as pneumonia, acute respiratory distress syndrome (ARDS), requiring intubation and emergency treatment. The virus binds to upper respiratory tract epithelial cells primarily through the ACE-2 receptor, which is highly expressed in adult nasal epithelial cells. The virus then undergoes replication and propagation within the upper respiratory tract, triggering the immune response responsible for the onset of typical symptomatology. If the immune response is not sufficient to contain the spread of the infection, lower respiratory tract (pulmonary alveoli) involvement and progression to acute respiratory distress syndrome (ARDS) occurs in severe cases [3]. Infected lung cells release a storm of cytokines (CS) that triggers an exaggerated host immune system response that can culminate in widespread cellular damage. As previously observed in other clinical conditions such as sepsis [4,5], the body’s immune response results in endothelial dysfunction that can induce microvascular damage, coagulation alterations, and consequently contribute to organ dysfunction [6]. In this regard, it has been reported that in COVID-19 patients, systemic microcirculatory changes accompanied by endothelial dysfunction correlate with the severity of ARDS [7]. The role of endothelial dysfunction is important considering that it has been associated with poor prognosis in the acute phase and with persistent symptoms, such as chest pain and fatigue, during the long COVID-19 period (4 weeks or more after onset infection) [8]. Therefore, an analysis of microcirculation and endothelial damage may play a key role in both the clinical course of COVID-19 and the evaluation of the long-term effects of this clinical condition. This evaluation could allow the development of new tools for monitoring patients to reduce the number of severe cases requiring intensive care units. In this context, the use of devices such as the pulse oximeter may be a valuable solution. The pulse oximeter is a non-invasive optical device based on the technique of photoplethysmography that allows the measurement of blood volume changes in a peripheral district, usually the fingertip or earlobe. The definition of the anatomical site where measurement is performed is a key point in the acquisition protocol, since perfusion characteristics vary according to the measurement location [9,10]. This device is commonly used for the estimation of heart rate and for the measurement of blood oxygenation (SpO2). In addition to these commonly monitored parameters, it is known that the characteristic components of the pulse oximeter waveform (PPG) are associated with specific circulatory functions [11,12]. In this perspective, a detailed analysis of the PPG waveform could provide important information on the microcirculatory function abnormalities and enable early recognition of patients with SARS-CoV-2 infection.

In our previous work, Rossi et. al [13], we investigated the feasibility of using the photoplethysmographic signal through a multi-exponential model to recognise patients hospitalised with COVID-19 and the severity of the disease itself. The photoplethysmographic signal was evaluated in 93 subjects with the aim of discriminating between healthy controls and COVID-19 patients of different severity. Using the parameters of the mathematical model, three different classifiers (Bayesian, SVM and KNN) were trained and tested, validating the results obtained by the leave-one-subject-out method. In this work, we will use the same dataset used in that study by proposing a different method for the analysis of the PPG signal. In particular, this article presents a new method for PPG signal pre-processing and a custom deep learning model that, starting from PPG signal analysis only, performs classification between COVID-19 patients and control subjects. Regarding the pre-processing phase, we developed a method that analyzes waveform morphology. Specifically, we adopted a Template Matching approach that performs a pulse-by-pulse comparison with a reference signal. With regard to the deep learning model, we developed a convolutional neural network architecture, a type of model that is finding increasing application in the field of biosignal analysis [14]. The method proposed in this paper, based only on the pulse oximeter signal, could be applied as a first assessment tool for the identification of COVID-19-induced microcirculatory alterations. Moreover, since the pulse oximeter is a low-cost device, as well as already widely used in hospital settings, the introduction of such a method would not imply any additional costs and could also be applied in healthcare settings with limited resources, such as those in underdeveloped countries and territorial emergency. Furthermore, due to the easy usage and the non-invasiveness of the device, this method could be useful in the development of a clinician-friendly system that could potentially be applied to other clinical conditions that have an impact on peripheral circulation, such as hypertension or sepsis.

The purpose of this study was to define a method, based on deep learning approaches, for the identification of COVID-19 subjects from the analysis of the raw PPG signal only. In addition, comparison with the results obtained with the different procedure [13] applied on the same sample of patients is a further objective of this study. In the Section 2, we reported the main studies that, similar to ours, have adopted a template-matching method for analysing the PPG signal or have used an artificial intelligence method applied to the photoplethysmogram. Our method is described in detail in Section 3. In particular, we described the data acquisition aspects, the implementation details of the pre-processing algorithm and the architecture of the neural network, together with the strategy adopted for the model training. The obtained results are reported in Section 4, while a discussion of them and a comparison with other work is given in Section 5, where limitations and future developments of the present study are highlighted.

## 2. Related Methods

There are many techniques that can be used to analyze the PPG signal. In this sense, knowing the performance and characteristics of different methods can contribute to optimising the treatment of patients. In this section, we report the main studies resulting from the literature review which, similarly to our method, adopted a template-matching approach for processing or an artificial intelligence approach for analysing the PPG signal.

Proposed template matching techniques differ from each other both in the method of reference signal (template) generation and in the metric used in the pulse-by-pulse comparison. Sukor et al. [15] derived a reference template by averaging the individual pulses of a PPG segment. The authors compared all pulses with the template by evaluating the Euclidean distance and the ratio between the amplitudes of the two signals. Acceptability thresholds for the two metrics were determined heuristically. Orphanidou et al. [16] and Karlen et al. [17] used Pearson’s correlation coefficient as a metric for the Template Matching. Orphanidou et al. derived the reference signal as the average of pulses in a PPG segment and then evaluated the correlation of each pulse with the template. The average correlation coefficient over the segment was then used as a metric for selecting good samples by imposing heuristic thresholds obtained from applying the method to different PPG sensors. Karlen et al., conversely, assessed the quality of each pulse by calculating the correlation between consecutive pulses, imposing a threshold for the correlation coefficient of 0.99. Considering a maximum number of consecutive pulses equal to 10 the assumption is that clean pulses taken from a short time interval are more or less equal to each other, unless they are corrupted with artefacts. Li et al. [18] used dynamic time warping (DTW) to match each beat to a template. By calculating the correlation and by using a signal clipping algorithm, the authors derived 4 features, which were used to train a multilayer perceptron with the goal of identifying good and bad-quality pulses. The DTW technique was also used in the study of Papini et al. [19]. The authors compared the morphology of PPG pulses with an adaptive template obtained by DTW barycenter averaging several beats, to consider physiological differences among individual pulses. The quality index of each sample was evaluated by taking into account the mean square error of dissimilarities between pulse and template. We recently proposed a PPG pre-processing method that required the generation of an ideal synthetic signal [20]. In particular, 3-s windows of the signal were compared with the reference signal by calculating the correlation coefficient. The use of a synthetic signal allows for very selective sample selection but it has the limitation of not accounting for the morphological variability of the waveform among the subjects.

Several studies used PPG waveform analysis applied to the study of cardiovascular disease. Nayan et al. [21] analyzed a set of 20 features extracted from the PPG signal using machine learning approaches for classification between healthy and COVID-19 subjects. The considered characteristics included amplitudes and time intervals of the main morphological features of the waveform: pulse onset, systolic peak, diastolic peak and dichrotic notch. The authors evaluated the performance of different classifiers, such as discriminant analysis (DA), k-nearest neighbour (KNN), decision tree (DT), support vector machine (SVM) and artificial neural network (ANN). The results obtained showed that ANN performed best in discriminating the two classes, achieving 95.45% of accuracy on the test set and 84.62% of accuracy on the validation set. Praveen et al. [22] used a feature vector extracted from the PPG signal to train three machine learning models (Random forest, Gradient boost, Xgboost) to classify blood pressure into 4 different stages of hypertension. Other approaches involve the use of deep learning methods to analyze the raw PPG signal, without requiring the process of feature selection and extraction from the data. Paviglianiti et al. [23] trained several neural networks to infer arterial blood pressure starting from photoplethysmogram (PPG) and electrocardiogram waveforms, obtaining good results on the estimation of diastolic and systolic pressure. Mahmud et al. [24] proposed a new approach for predicting the severity of hypoxia using deep learning applied to the PPG signal. This method is an alternative to the traditional application of the pulse oximeter, which, having a high sensitivity in detecting oxygen degradation, often has a high rate of false positives that could lead to desensitization of healthcare operators.

## 3. Materials and Methods

### 3.1. Data Acquisition

Data acquisition was carried out at S. Giuseppe Hospital in Empoli, Italy. A total of 183 subjects were recruited for the study, including 93 subjects affected by COVID-19 and 90 control, healthy subjects not affected by the target disease. The COVID-19 group included RT-PCR-positive subjects admitted to the hospital with medium to high disease severity, identified by the need for treatment with a high-flow nasal cannula (HFNC) or noninvasive ventilation (NIV). Subjects in the control group were recruited from the hospital’s healthcare staff including healthy subjects not affected by COVID-19 or by other cardiovascular diseases. Only subjects older than 18 years and of white Caucasian ethnicity were included. The inclusion criterion on ethnicity resulted from the fact that as shown in recent studies, many factors can influence the PPG waveform, and among them one of the most significant is the skin color [25,26]. All participants accepted informed consent before being enrolled into the study.

The patient cohort recruited for the study was the same as the one used in the work of Rossi et al. [13] except for the number of control patients, which was increased to balance the number of subjects with COVID-19. Among the covid group, 64% of subjects were men and 36% were women, while in the control group, men accounted for 37% of subjects and women for 63%. The mean and standard deviation of age were (65.93 ± 17.75) for septic subjects and (43.99 ± 11.16) for control subjects.

For each subject, the protocol consisted of the acquisition of the photoplethysmographic trace using a finger pulse oximeter. The acquisition took place under resting conditions and for a duration of at least 5 min. The measurement site was the index finger of the right hand for all subjects involved. The acquisition system consisted of a finger pulse oximeter connected to the Mindray ePM-10 monitor, commonly used in the hospital for continuous monitoring of patients’ vital parameters. A Raspberry Pi 3 device, connected to the monitor using a network connection and an HL7 (Health level seven) protocol, was used to store the waveforms. Data were acquired with a 60 Hz sampling frequency and stored as standard HL7 messages. In the first decoding step, PPG waveform values were extracted from the HL7 message for each subject. Then the signals were stored with a progressive numerical code so as to eliminate any identifying data that could trace back to the patient.

### 3.2. PPG Quality Assessment

The PPG waveform is susceptible to various forms of noise. Among these, one of the most common is the presence of motion artefacts that distort the shape of the signal. In this study, we developed an algorithm for the evaluation of PPG signal quality based on waveform morphology. A PPG pulse is characterised by a rising phase (anacrotic phase), which represents the systolic phase of the heart, and a falling phase (catacrotic phase), which represents the diastolic phase. A valley, called a dichrotic notch is often present in the catacrotic phase of the waveform, and is associated with aortic valve closure and good arterial function [27]. The use of morphological features to assess PPG signal quality has been widely used in the literature. One of the most common methods is a pulse-by-pulse comparison with a reference signal, which is called Template Matching. In this work, we implemented a Template Matching method by deriving, from each acquisition, a patient-specific reference pulse. This pulse was then compared with the entire PPG signal through the calculation of the Pearson correlation coefficient. The good quality portions of the signal were selected by imposing a threshold for the correlation coefficient.

#### 3.2.1. Template Calculation

**Figure 1:**
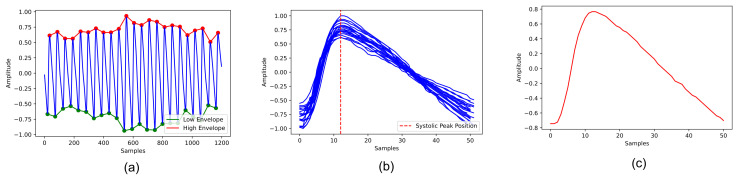
Main steps for calculating the reference template for each patient. (**a**) shows the lower and upper envelope of the signal. (**b**) shows the alignment of segmented pulses on the systolic peak. The calculated template is presented in the (**c**).

In our study, each patient-acquired signal was processed to obtain a specific reference pulse. Each PPG acquisition was normalised to have values between −1 and 1, and then the signal was filtered with a Butterworth bandpass filter with cutoff frequencies of 0.5 and 5 Hz [15]. The filtering allowed the preservation of spectral components related to cardiac activity, thus, facilitating subsequent identification of systolic peaks. From the filtered PPG, the lower and upper envelope of the signal were calculated to identify the position of the pulse onset and systolic peaks (Figure 1a).

This allowed the segmentation of each individual pulse of the signal, identified as the waveform between two consecutive onsets. At this stage we provided limits to the pulse duration imposed by natural cardiovascular physiology so that only those peaks that met the physiological limits were considered for template calculation. Specifically, the limits imposed include minimum and maximum values for the systolic phase (SP), that is the rising wave between the pulse onset and the systolic peak, and limits for the pulse wave duration (PWD). The acceptable values for the duration of the systolic phase were in the range of 0.08 to 0.49 s, as described in the study of Fisher et al. [28]. The constraints for PWD were calculated, as described in Equation (1), by imposing a minimum mean heart rate of 40 bpm and a maximum mean heart rate of 180 bpm, considering that the subject was in a resting state during acquisition.

(1) $$PWD_{min}=\frac{60\times F_{s}}{HR_{max}};\;PWD_{max}=\frac{60\times F_{s}}{HR_{min}}$$

**Figure 2:**
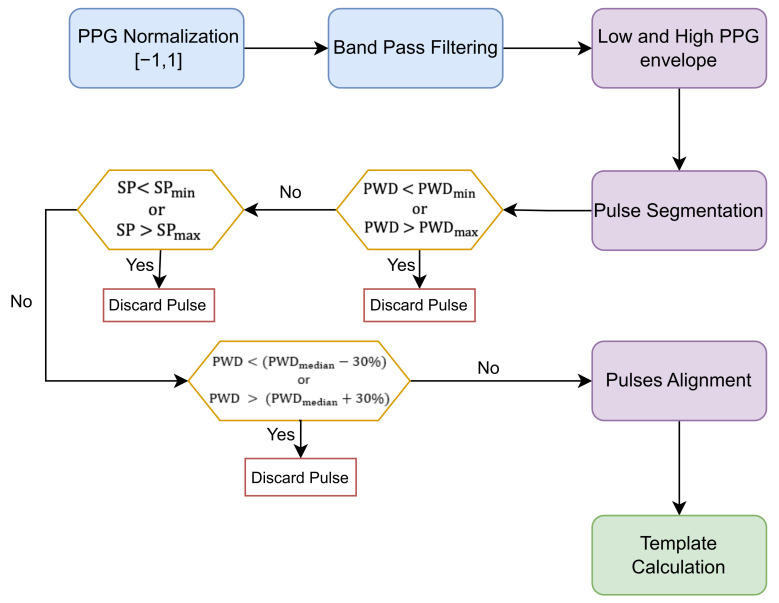
Flowchart of algorithm for template generation.

With regard to the pulse duration, we also derived a PWD reference value by calculating the median of the width of the pulses. Samples with PWD that differed from the median value of more than 30% were not considered for template calculation. The selected pulses were then aligned on the systolic peak, as shown in Figure 1b. The reference point for alignment was calculated as the mean of the position of the systolic peak of all pulses. To obtain pulses of the same length, we performed truncation of the longer samples and constant-value padding at the beginning or end of the signal for the shorter samples. Once the samples were aligned, we obtained the template by calculating the median of the pulse waveforms (Figure 1c). The implemented algorithm for template calculation is summarised in Figure 2

#### 3.2.2. Quality Assessment

Once the reference template was obtained for each patient, signal quality was assessed by calculating the Person’s correlation between the template and each segmented pulse of the patient acquisition. Each pulse was rated of acceptable quality if it correlated with the template equal to or greater than 0.8. The threshold for the correlation coefficient was determined empirically by visual inspection of the waveforms. Therefore, we stored all portions of the signal that contained consecutive pulses labelled as being of good quality. As a result, we obtained PPG samples of varying lengths associated with the same subject. Among these, we only selected for further analysis those samples with a minimum duration of 30 s. The minimum duration of 30 s was chosen experimentally, considering the need to select a waveform of the longest possible duration and the need to have as much data as possible available for training the neural network. As a result of the preprocessing algorithm, we obtained 336 PPG samples. Specifically, 186 samples from 81 patients of the control group, while a total of 150 samples from 84 patients from the covid group.

### 3.3. Dataset Construction

The selected PPG samples were then divided into a training set and test set. As a result of the pre-processing algorithm, multiple PPG samples could be associated with each patient. The division of the available data was done to ensure that data from a specific patient was present in only one of the two sets. The assignment of subjects to the training or test set was done completely randomly. The training set was used for neural network model development, while the test set was used for model performance evaluation. Since the PPG samples can have variable durations, we segmented the test samples to obtain a fixed set of PPG segments on which to perform neural network performance evaluation. Segmentation was performed by deriving for each PPG sample all possible 30-s duration windows from the onset points of individual pulses. The number of samples and the number of subjects in each set of data are reported in Table 1.

### 3.4. Neural Network Architecture

**Figure 3:**
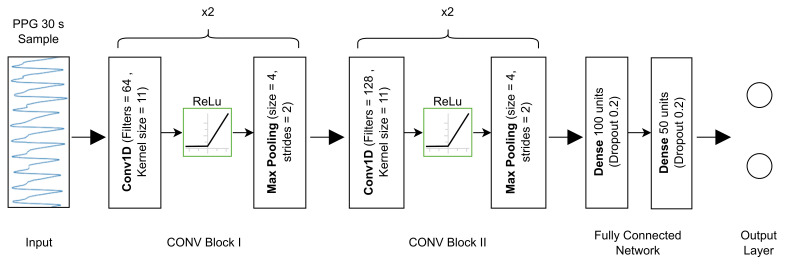
Description of our custom CNN architecture.

The design, training and testing of the neural network were implemented in Python using the Tensorflow and Keras frameworks. All experiments were conducted on a computer with an Intel i9-11900 2.5 GHz processor and 48 GB RAM within the Microsoft Windows 10 Pro operating system (Lenovo Italy S.R.L., 20054 Milano, Italy). The model structure used in this study is an architecture based on a convolutional neural network (CNN). CNN architectures are made of 3 main layers: the convolution layer, the pooling layer and the fully connected dense layer. The convolution layers and pooling layers compose the first block of the model, which is devoted to featuring extraction from the input data. The last block of the architecture consists of a fully connected network formed by dense layers, and is responsible for associating the extracted features with the desired output. Our custom model consists of 4 feature extraction blocks (CONV Block), each comprising a 1D Convolution layer, ReLu activation and a Max Pooling layer. The first two CONV Blocks have a number of filters equal to 64, while in the last two, the number of filters is 128. All convolution layers have a kernel size of 11 and all Max Pooling layers have a filter width of 4 and stride size of 2. The fully connected network includes a first dense layer with 100 units, followed by a layer with 50 units. For both layers, we included the dropout method with a rate of 0.2 as a regularization strategy to prevent model overfitting. The output layer contains two nodes with softmax activation, as we want to discriminate between two classes. As input, the model takes 30-s PPG segments normalised to have values in the range [−1, +1]. The detailed description of the proposed architecture is shown in Figure 3.

Regarding the complexity of the proposed model, we analyzed some of the most commonly used metrics to assess the complexity of artificial neural networks: the number of trainable parameters, the number of Floating Point Operations (FLOP) and the inference time. As for the first metric, our model has 1,614,532 trainable parameters. With regard to the number of FLOP, this metric represents the total number of calculations (for example, additions or multiplications) that the model has to perform to process an input sample. Each layer of the model involves performing a number of operations that depend on the structure of the layer itself, e.g., the number of FLOP for a one-dimensional convolutional layer depends on the number of filters, the kernel size, the number of input features and the output size. For our architecture, we estimated the number of floating-point operations equal to 236.74 MFLOP using the TensorFlow Python API. Finally, the inference time represents how long it takes to process an input and produce the output. This parameter depends on the available hardware and, in particular, on the number of Floating Point Operations per Second (FLOPS). This measure can be obtained from the CPU specification and, in our case, is 3.2 × 10^5^ MFLOPS. The inference time was then calculated by dividing the number of FLOP required from the model by the number of operations per second supported by the CPU, yielding an inference time of 0.74 ms.

### 3.5. Model Training

When working with neural networks, three sets of data are usually used for training, validation and testing of the model, respectively. At the same time, to evaluate the generalization ability of the model, cross-validation is typically adopted. There are several ways to validate a model, in this case, we adopted 5-fold cross-validation. Validation data were derived from the training set by performing stratified group sampling, where each group contains PPG samples related to a specific patient. In this way, we obtained 5 sets of PPG samples containing data from different subjects, thus, permitting evaluation of the robustness of the method with respect to data variation. In each iteration, 1 of the 5 groups constituted the validation set, and the other 4 were used to train the model. The same architecture, previously described in Figure 3, was used for each cross-validation iteration.

Our architecture takes 30-s PPG segments as input examples. Therefore, a segment of the desired duration was derived from each sample in the training set. The selection of that segment was made during the training process by considering a 30-s window that had as its starting point the onset of one of the individual pulses that constitute the waveform. At each iteration, the selected window was different; thus, the model was trained with many different portions of a signal from the same patient. The range of values assumed by each input data was between −1 and 1.

Regarding the selection of the training hyperparameters, a trial-and-error approach was used, evaluating the trend of the learning curves and the performance obtained by the model on the validation and test set. The investigated parameters were batch size, learning rate, optimiser, loss function and the number of epochs. The chosen parameters for the final version of the model are summarised in Table 2.

Given the limited amount of data available, after validating our method in cross-validation, we re-trained the model using all the data in the training set, assuming to improve the performance due to the utilization of more data.

## 4. Evaluation Results

The model was evaluated in the training phase by considering the average performance obtained on the cross-validation sets and then on the data selected for the test set. In both cases, the evaluated metrics were: accuracy, sensitivity, specificity and precision. Areas under the curve (AUC) of the Receiver Operating Characteristic (ROC) curve and the Precision-Recall (PR) curve were also measured.

The cross-validation process produced 5 different models. Each model differed from the other in the subjects used in training and validation, thus, permitting assessment of the robustness of the method with respect to the physiological variability of the subjects. The average performance of our architecture on the validation sets resulted in an accuracy of 79.01%, a sensitivity of 80.02%, a specificity of 76.57% and a precision of 74.95%.

Then the performance of each model was evaluated on the test set data. All models showed consistent performance on test data, as shown in Table 3. In addition to the average performance of the models, we evaluated an “ensemble” approach, previously used in our other work [29], in which all models were combined in the prediction process. In this method, for each test sample, all models were consulted and the class obtaining the majority of votes was considered as the final prediction.

**Figure 4:**
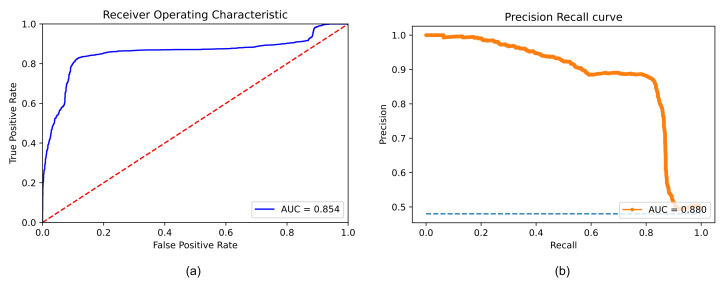
Model performance on the test set. (**a**) shows the ROC Curve and the correspondent AUC. (**b**) illustrates the PR and the associated AUC. Each point, on both curves, is derived from the values of the confusion matrix associated with the application of a specific cutoff to the predictions of the classifier.

As we expected, the combined use of the 5 models resulted in an increase in performance over that achieved by a single one. Similarly, we evaluated the performance on the test set after using all the training set data to train the neural network (hold-out validation). The obtained results are summarised by the ROC Curve and the PR Curve shown in Figure 4.

**Figure 5:**
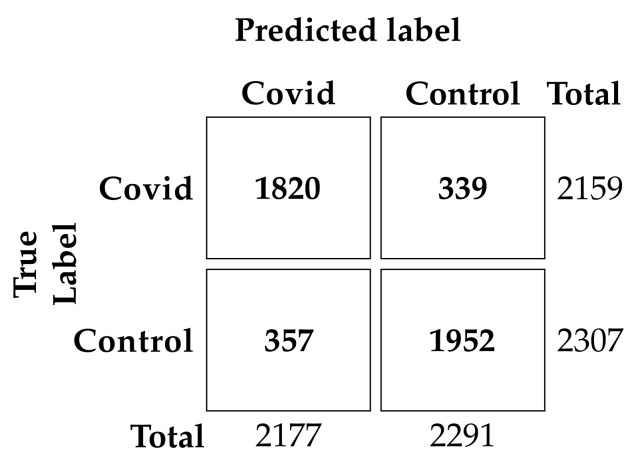
Confusion matrix on the test set using the hold-out validation.

These curves show the ability of a model to classify binary outcomes for each possible cutoff value applied to the classifier’s predictions. Specifically, the ROC curve is generated by plotting a model’s false positive rate against the true positive rate, while the PR curve plots the true positive rate (recall or sensitivity) against the positive predictive value (precision). With a threshold equal to 0.5, our model achieved an accuracy of 83.86%, a sensitivity of 84.30%, a specificity of 83.45% and a precision of 82.46%. The total number of predictions for each class is described in the confusion matrix, shown in Figure 5.

The results obtained in the hold-out validation confirm our hypothesis that more data available for model implementation could lead to improved performance.

The most significant parameter for our study is sensitivity, which identifies the percentage of COVID-19 samples correctly identified. This parameter reached a good value of 84.30%. However, since multiple 30-s PPG windows are associated with each subject, to assess the true percentage of correctly classified subjects, we performed the test on the individual patient. In this case, we evaluated the number of correctly identified PPG samples for each patient. Therefore, each subject was considered to be correctly classified if most of his/her signal samples were associated with the right class. In this testing modality, our method correctly classified 25 of the 32 patients assigned to the test set, corresponding to an accuracy of 78%, sensitivity of 75% and specificity of 81%.

## 5. Discussion

In this study, we evaluated the possibility of using the PPG signal to identify patients infected with COVID-19. Specifically, we presented a new template matching method for PPG signal pre-processing and we developed a CNN deep learning-based model for analyzing the photoplethysmographic signal acquired with a common pulse oximeter. Data acquisition was carried out at S. Giuseppe Hospital in Empoli, using the Mindray multiparameter monitor commonly used in the Intensive Care Unit, thus, simulating a real application of the developed method. The collected data were divided into training set and test set, which were, respectively, used for classifier training and performance evaluation. To assess the robustness of the classifier with respect to variation in the subjects used for performance evaluation, we initially implemented cross-validation and then performed hold-out validation. In the hold-out validation, our model showed good performance in the classification between COVID-19 patients and control subjects by achieving an accuracy of 83.86% and a sensitivity of 84.30% on the test data. Observing the ROC curve related to our classifier (Figure 4), it can be seen that the curve reaches a plateau. This means that as the threshold applied to the model’s predictions increases, there is no corresponding improvement in the performance of the classifier. Therefore, it can be deduced that there are some patients whom the model fails to classify. We can hypothesise that this performance may be due to unimpaired microcirculation in these subjects.

Overall, the obtained results confirmed the presence of microvascular changes due to SARS-CoV-2 infection and the potential of photoplethysmography as a tool for microcirculation assessment. This method, based only on samples of the PPG signal, seems to be suitable for a rapid screening procedure, which could provide the clinician with an early warning signal and allow, for example, the use of specific diagnostic procedures. Moreover, given the noninvasiveness and wide use of this device, especially in the hospital setting, this method could be a tool for the evaluation of microcirculatory changes that does not introduce additional costs. The results obtained allow us to compare the usefulness of using deep learning approaches versus other methods based on feature extraction from the photoplethysmogram. In the study of Nayan et al. [21] a set of features extracted from the PPG signal was used to classify COVID-19 patients using Machine learning approaches. Similar to our study, the classifiers were trained by implementing a 5-fold cross-validation on the training data and then evaluated on the test set. In particular, the best performing classifier was a feed-forward multilayer perceptron network, which achieved consistent performance on both the validation set and the test set, in contrast to other classifiers that had significantly lower performance on the validation set. The authors obtained excellent results achieving more than 90% accuracy on the test set and 84.62% accuracy on the validation set. Although our work yielded lower performance than the method described by Nayan et al. we believe it still has advantages. Differently from that study, our method does not require the process of extracting and selecting morphological features from the PPG signal, but processes 30-s windows of the raw PPG signal. This could be particularly advantageous in the case of signals acquired under uncontrolled conditions, such as those acquired from multi-parameter monitors in Intensive Care Units, for which the extraction of PPG features could be challenging. In our previous study, Rossi et al. [13], the PPG features were derived by fitting the waveform with a 3-exponential model. The model parameters were then used in ML approaches to identify COVID-19 patients with different severity. Since this study is based on the same data set, the aim of this study is to compare the obtained results with those achieved using the exponential photoplethysmogram model. Given the limited number of subjects enrolled in the study and, consequently, the limited amount of data available for training the neural network, our work focused on the classification between control healthy subjects, indicated as group 0, and covid subjects, regardless of severity, identified as a group (1, 2) in our previous study. In that study, the comparison between group 0 and group (1, 2) was performed in three different ways utilising the Bayesian Classifier with the Leave-One-Subject-Out (LOSO) validation method. The classifier was trained both with features extracted from a single beat and with features averaged over two consecutive beats. The classification of the patient was then obtained based on the majority of the classifications of the single or pairs of cycles. Furthermore, performances were evaluated by considering a single feature vector per patient, obtained by averaging the characteristics over the entire acquisition. The best performance was obtained using the average feature vector, resulting in an accuracy of 70%, sensitivity of 68% and specificity of 74% in the classification of subjects. Although the methods are not directly comparable, as they were validated using different methods, we are interested in comparing the performance of the two approaches in terms of correctly classified subjects. In this respect, we can observe that the method proposed in this work, based on a deep learning model, performed better in classifying individual subjects, achieving an accuracy of 78%, a sensitivity of 75% and a specificity of 81%.

### Study Limitations and Future Developments

Overall, the findings confirm the potential of the proposed method for the early assessment of microcirculation alterations in COVID-19 patients. However, it is necessary to consider some limitations of this work that open the way for further investigation and development of the implemented method. The main limitations are related to the dataset used. The data for training and evaluation of the model were all acquired in the same hospital. To assess the generalization ability of the model, we plan to evaluate its performance on at least one other database. In addition, we hypothesise that a greater number of data available may improve the performance of the model, as well as allow for an evaluation of performance on a larger population. Finally, the two groups of subjects enrolled in the study, although balanced in number, are biased in terms of gender and age. Investigating the influence of these two parameters on the performance of our method will be our further goal. In this regard, we are aware that interpretability of the model is a fundamental requirement for applying this method in the medical field. For this reason, further validations will be necessary to make the model explainable and consequently improve the clinician confidence in using this method. Finally, given the potential shown in this work by the photoplethysmographic technique in the evaluation of microcirculatory alterations, in our future work, we are interested in exploring the use of PPG imaging since optical imaging techniques may also allow a description of the spatial distribution of peripheral blood flow [30,31].

## 6. Conclusions

In this study, we evaluated the possibility of using the PPG signal for the screening and classification of patients with COVID-19. Specifically, we developed a custom convolutional neural network model that discriminates between Covid patients and control subjects by analyzing only the PPG signal. The proposed method achieved interesting results in terms of accuracy (78%), sensitivity (75%) and specificity (81%) on the test set data. Overall this study confirms that PPG signal may be used for the screening of patients with COVID-19 and the assessment of microcirculatory alterations. Moreover, these results are important because acquiring the photoplethysmographic trace is simple, noninvasive and inexpensive. In this regard, this method could be used to develop a user-friendly system that could represent an initial assessment tool for the clinician, applicable even in clinical settings with limited resources. Further studies with a larger sample size of patients and with data from other databases, as well as an evaluation of the interpretability of the model, will be needed to assess the effectiveness of the proposed method.

## Figures and Tables

**Table 1:** Description of training and test sets data.

	**Training Set**		**Test Set**		**Test Set Segmented**	
	**COVID**	**Control**	**COVID**	**Control**	**COVID**	**Control**
**N subjects**	68	65	16	16	16	16
**N samples**	119	143	29	36	2159	2339
**Ratio [%]**	45.4%	54.6%	44.6%	55.4%	48.0%	52.0%

**Table 2:** Chosen hyperparameters for model training.

Hyperparameter	Value
Learning rate	1 × 10^−6^
Number of Epochs	800
Batch size	8
Optimiser	Adam
Loss Function	Mean Squared Error (MSE)

**Table 3:** Performances of 5-fold cross-validation sets on test data.

	Accuracy [%]	Sensitivity [%]	Specificity [%]	Precision [%]	AUC ROC	AUC PR
**Fold I**	83.95	87.45	80.72	80.72	0.85	0.88
**Fold II**	82.33	83.97	80.80	80.15	0.84	0.84
**Fold III**	78.21	81.15	75.50	75.35	0.82	0.84
**Fold IV**	80.50	81.24	79.82	78.80	0.81	0.80
**Fold V**	83.06	83.00	83.11	81.94	0.83	0.81
**Average Performances**	81.61	83.36	79.99	79.39	0.83	0.83
**Model Ensemble**	82.88	85.27	80.68	80.29	0.84	0.86

Each fold of data differs in the subjects used in training and validation. Each fold produced a different trained model, whose evaluation on the test set data is shown in the table. The table reports both the performance of the individual models, the average performance and the performance obtained using the ensemble of models.

## Data Availability

Not applicable.
